# Effect of vertical, horizontal, and combined plyometric training on jump, sprint and change of direction performance in male soccer players

**DOI:** 10.1371/journal.pone.0295786

**Published:** 2024-05-23

**Authors:** Jason Moran, Norodin Vali, Anders Sand, Marco Beato, Raouf Hammami, Rodrigo Ramirez-Campillo, Helmi Chaabene, Gavin Sandercock

**Affiliations:** 1 School of Sport, Rehabilitation and Exercise Sciences, University of Essex, Colchester, United Kingdom; 2 Department of Exercise Physiology, Shahid Rajaee Teacher Training University, Tehran, Iran; 3 Division of Speech and Language Pathology, Department of Clinical Science, Intervention and Technology, Karolinska Institutet, Stockholm, Sweden; 4 School of Health and Sports Sciences, University of Suffolk, Ipswich, United Kingdom; 5 Higher Institute of Sport and Physical Education of Ksar-Said, Universite de La Manouba, Tunis, Tunisia; 6 Research Laboratory: Education, Motor Skills, Sports and Health (EM2S, UR15JS01), Higher Institute of Sport and Physical Education of Sfax, University of Sfax, Sfax, Tunisia; 7 Exercise and Rehabilitation Sciences Institute, School of Physical Therapy, Faculty of Rehabilitation Sciences, Universidad Andres Bello, Santiago, Chile; 8 Division of Training and Movement Sciences, Research Focus Cognition Sciences, University of Potsdam, Potsdam, Germany; 9 Higher Institute of Sports and Physical Education, Kef, University of Jendouba, Jendouba, Tunisia; Jerzy Kukuczka Academy of Physical Education In Katowice: Akademia Wychowania Fizycznego imienia Jerzego Kukuczki w Katowicach, POLAND

## Abstract

**Purpose:**

The purpose of this study was to compare the effects of vertical (VPT), horizontal (HPT) and combined vertical and horizontal (V+HPT) plyometric training on sprint, jump and change of direction (COD) performance in adult male soccer players.

**Method:**

Participants were randomly allocated into VPT (n = 8), HPT (n = 8) and V+HPT (n = 8) groups which undertook eight weeks of PT, executing 100 foot contacts per session, twice weekly.

**Results:**

Though demonstrably effective, no specific one of the three applied programmes enhanced performance to a greater extent than another with only the 40 m sprint for the HPT group (mean difference = 0.07 s [HPT] vs. 0.04 s [VPT] and 0.04 s [V+HPT]) and the vertical jump for the V+HPT group (mean difference = 4.5 cm [V+HPT] vs. 4.0 cm [VPT] and 3.25 cm [HPT]) appearing to deviate from a uniform pattern of group level adaptation across the performance tests.

**Conclusion:**

A total volume of 100 foot contacts per session, twice per week for eight weeks was sufficient to achieve the observed changes. Though jump and changing direction performance were enhanced, linear sprint performance was largely unchanged and so a more complete and intense programme may have been warranted. No method was superior to another in eliciting changes across these tests and a directionally-specific pattern of adaptation was not apparent.

## Introduction

An athlete’s ability to generate force at specific velocities, and in specific directions, is important in sports that require dynamic actions such as jumping or sprinting [1]. The training specificity principle supports the use of vertical and horizontal jumps to improve performance in vertically- and horizontally-orientated tasks such as jumping and sprinting respectively [2, 3]. In strength and conditioning terms, this principle is underpinned by the theory of dynamic correspondence which serves as an accepted framework for the development of sport-specific programmes of physical preparation [3–5]. In this way, a jump executed in a predominantly horizontal direction might be more effective than a vertical jump for enhancing sprint speed; whereas, a vertical jump may be more effective in enhancing a biomechanically-similar skill such as a spike jump in volleyball [5]. Accordingly, this informs the selection of training methods that a coach can choose to optimise athletes’ performance [6, 7].

A recent meta-analysis [5] summarised the literature on the effects of vertically- and horizontally-orientated plyometric training (PT) on directionally-specific athletic performance. It found that whilst both horizontal PT (HPT) and vertical PT (VPT) were both effective for enhancing jump and sprint performance, HPT was just as effective as VPT in enhancing vertical performance (i.e. vertical jump) but was superior at enhancing horizontal performance (i.e. horizontal jump, or sprint). Therefore, compared to VPT, a HPT approach would appear to be the more effective training activity as it seemed to induce similar vertically-orientated performance while eliciting greater horizontally-orientated performance. The authors of the review [5] suggested that this could be due to the characteristics of horizontal and vertical jumps. For example, vertical jumps exhibit no displacement of a performer’s centre of mass in a horizontal direction at take-off [5, 8]. However, horizontal jumps demonstrate displacement of the centre of mass both horizontally and vertically suggesting that there is a horizontal and a vertical component to horizontal jumps, but only a vertical component to vertical jumps [5, 8].

Though the results of the aforementioned meta-analysis [5] summarise the known evidence on the topic of VPT’s and HPT’s effect on athletic performance, this still represents a relatively small body of literature, thus necessitating further investigations. Two recent studies [9, 10] have provided additional knowledge in this area since the meta-analysis of Moran et al. [5] but none since the intervention of Ramirez-Campillo et al. [11] in 2015 have compared the effects of VPT, HPT and combined VPT and HPT (V+HPT) on athletic performance, while no study of this type has ever been carried out in an adult population. Due to this shortcoming in the literature, it is not entirely clear how coaches should approach the prescription of HPT and VPT, and their combination, in an athletic adult population, a surprising deficit in a body of research that has grown rapidly in recent years. The purpose of our study was therefore to address the gap in the literature by comparing the relative effects on sprint, jump and change of direction (COD) performance of VPT, HPT and V+HPT in adult male soccer players.

## Methods

### Experimental approach

The study took place from May to July, 2022, starting ten weeks into the soccer season before which the players undertook a pre-season training period. This pre-season training was not part of the current study but, for informational purposes, it comprised five weeks during which players participated in six to seven training sessions per week. One session was devoted to circuit-style strength training which included barbell squats, barbell chest presses, walking lunges and modified pull-ups, as well as the flat and side plank exercises. In other sessions, the main focus was on improving tactical skills, technique and cardiovascular fitness with small sided games, interval-based running and speed and agility exercises used to enhance the physical fitness of the players for the upcoming season.

The training intervention was carried out over a period of eight weeks, the composition of which can be seen in Table 1. The exercises were chosen on the basis of their directional orientation according to the principle of training specificity. The approach was based on previous meta-analysis [5] that supported the superiority of HPT but recommended further comparison with VPT and especially a combination of both, over a period of longer than seven weeks. The specific training parameters followed those recommended for soccer players by Ramirez-Campillo et al. spanning a period of more than seven weeks, incorporating two sessions per week, and a volume of 140–240 jumps per week (200 jumps were executed weekly). Based on those recommendations, the jumps were performed with maximal effort, utilising correct technique and a rest interval that exceeded 30 seconds between sets being observed (60 to 90 seconds was used). Additionally, recovery between sessions was around 48 hours. Players were randomised into groups which performed VPT, HPT and V+HPT twice per week. The training intervention was executed on Thursdays and Saturdays. Before and after the intervention, players were tested in 10 m and 40 m sprint, 505 COD, standing long jump (SLJ) and vertical jump (VJ). The study was approved by the university ethics committee and conformed to the Declaration of Helsinki.

**Table 1:** Training intervention.

	Vertical group		Horizontal group		Vertical+horizontal group	
	Sets x repetitions	Total volume	Sets x repetitions	Total volume	Sets x repetitions	Total volume
Horizontal ankle hops	n/a		5×12	60	5×6	30
Horizontal long jumps	n/a		5×8	40	5×4	20
Vertical ankle hops	5×12	60	n/a		5×6	30
Vertical jumps	5×8	40	n/a		5×4	20
		100		100		100

The players undertook six training sessions in total each week. The day after a match, which occurred on a Monday, the players rested while those who played less than 70 minutes engaged in high-intensity interval training and small-sided games. On Wednesday, all players participated in a recovery session. On Thursday, following a comprehensive warm-up, players were divided into three groups and performed the PT research protocol simultaneously. After carrying out the PT, the players jointly participated in a soccer training session which included technical and tactical exercises. On Friday, the players jointly participated in further technical and physical training. Saturday, once again after a warm up, each group performed the PT protocol. On Sunday, an activation protocol was carried out by all players. All sessions for all of the experimental groups were overseen and supervised by a strength and conditioning coach who provided extensive direction on the performance of the exercises and controlled the application of the training load.

### Participants

Twenty-four semi-professional soccer players (age: 22.3 ± 2.7 years; height: 181.7 ± 6.2 cm; body mass: 73.7 ± 7.2 kg; BMI: 22.3 ± 1.0 kg/m^2^; soccer training experience: 11.2 ± 2.8 years; resistance training experience: 4.5 ± 2.0 years) from the same team (Iranian tier 3 league) participated in this study. The players were recruited in June, 2022. They were randomly divided into three training groups: HPT (n = 8), VPT (n = 8) and V+HPT (n = 8). No goalkeepers took part in the study.

### Procedures

Players were asked to follow their regular diet on the day of the assessments of athletic performance and to not consume any stimulants. Prior to each assessment, the players were given 48 hours of rest. The assessments were always performed on the same day of the week (Saturday, Monday and Wednesday) between 6 pm and 7 pm. The players wore their usual soccer footwear and performed the tests on the natural grass surface that they were accustomed to playing on. The warm-up for the tests was the FIFA11+ protocol [12], executed with minor changes. All players were fully acquainted with the utilised athletic performance tests having being familiarised with them through their previous training activities. The order of the assessments was as follows: on day one, anthropometric measurements were undertaken including height, body mass and body fat percentage. On day two, the 505 test was used to evaluate COD ability. On day three, the participants undertook the jump tests. On day four, 10 m and 40 m sprint speed were measured. The rest interval between each effort in each of the various tests was three to five minutes.

#### Anthropometry

Stature and body mass were assessed between 8 am and 10 am on the first day of testing. The assessments were made by the same observer. Stature was assessed using a stadiometer (Seca 217 Stable stadiometer, Hamburg, Germany) and body mass was measured using an accompanying scales.

#### Horizontal jump

The SLJ was employed to measure horizontal jumping performance and followed the protocol of previous researchers (intraclass correlation coefficient [ICC] = 0.94) [13]. Participants were guided through an initial familiarisation trial during which the key aspects of execution were communicated to them. The participants stood behind a line marked on the ground with feet slightly apart and were asked to maintain a parallel foot position during both take-off and landing of the jump. The jump was measured using a long jump mat (Jump length pairs, Tanazma, Iran) marked in centimetres. The jump distance was recorded corresponding to the position of the heels to the nearest point of contact upon landing. Each participant was permitted two trials with the best performance being used for further data analysis.

#### Vertical jump

The Sargent Jump Test was used to gauge VJ performance. A familiarisation trial was first carried out along with identification of the key technique aspects of the movement. The VJ was measured using a tape measure attached to the wall. Participants were measured standing side onto this wall whilst reaching upward as high as possible with the tips of their fingers. To execute the jump, participants started in a standing position and descended into a flexed-knee position to a depth of their choosing before jumping as high as possible and marking the wall with the chalk. The VJ was measured according to the distance from the aforementioned standing position to the mark made during the jump. When executing the VJ, participants were not allowed to stop the movement during the descent or propulsive phases. Each participant was assessed twice with a passive rest period between efforts. The best performance of these two jumps (cm) was recorded for further analysis. The intra-class coefficient (ICC) in this test was 0.88 [14].

#### Sprint test

To measure sprinting speed, electronic timing gates were used (Newtest Powertimer 300-series testing system, Finland). This test has been shown to be highly reliable in the measurement of linear sprint speed (ICC = 0.89–0.9) in soccer players of a similar age [15, 16]. Distances of 10 m and 40 m were used to determine the sprinting speed of the players. Participants started in a split-legged stance with the preferred foot positioned 70 cm behind the first pair of photocells that marked the starting line. Three pairs of photocells were used (starting line, 10 m and 40 m). The photocells were positioned at roughly hip height to enable the capturing of trunk movement, rather than a false trigger from a limb. Players performed two trials with the fastest used for further data analysis.

#### Change-of-direction test

The 505 test protocol was employed to measure the COD speed of the players. Electronic timing gates were used (Newtest Powertimer 300-series testing system, Finland). This test has been shown to be highly reliable in the measurement of COD ability (ICC = 0.93) in soccer players of a similar age [16]. From a split-stance starting position, the participants were required to sprint 5 m before touching their foot on the demarcated line and then performing a 180-degree turn, positioning their body to sprint 5 m back through the starting point. The players were allowed to use their preferred leg for braking and turning, however, they were asked to use the same leg for each effort. The photocells were positioned at hip height to enable the capturing of trunk movement rather than a false trigger from a limb. Players performed two trials with the fastest used for further data analysis.

### Statistical analyses

#### Data analysis

We graphically illustrated the training response trajectories for each individual participant in the different training tasks and visually compared the response trajectories in the three training groups (VG, HG, VHG). We also calculated improvement scores for the different training tasks. For the sprint tests, we calculated improvement by subtracting the follow-up score from the baseline score such that *improved* sprint performance is measured by a positive number of seconds. For the jumping tests, we calculated improvement by subtracting the baseline score from the follow-up score such that *improved* jump performance is measured by a positive number of centimetres. We further compared the three training groups by calculating and comparing the mean and median improvement score in each group and training test. Of note, we did not compare the training groups based on null hypothesis tests as *p*-values *do not* indicate whether “there is an effect”, or not, in a sample [17, 18]. For informational rather than analytical purposes, we also present group effect sizes (Cohen’s *d*) alongside 95% confidence intervals (CI).

## Results

**Fig 1:**
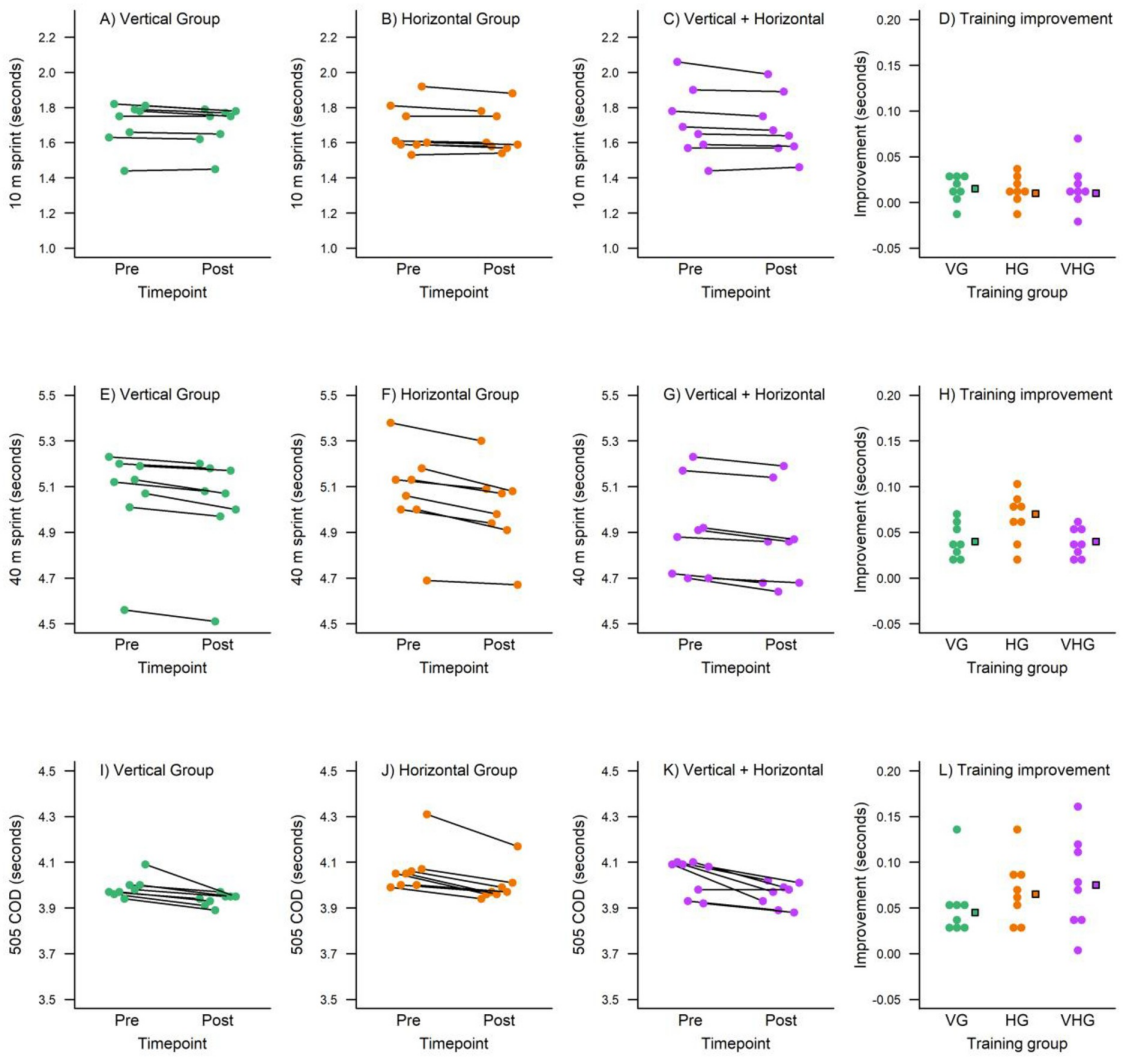
Descriptive plots for the training measures at the 10 m sprint (top row), 40 m sprint (middle row), and 505 COD test (bottom row) separate for the three training groups (columns). The VG is illustrated in turquoise, HG in orange, and VHG in violet. In the rightmost panels (panel D, H, and L) is the training improvement (*pre* minus *post*) for each individual participant (circles). The median improvement in each training group is also illustrated in the rightmost panels as a square in matching colour.

**Fig 2:**
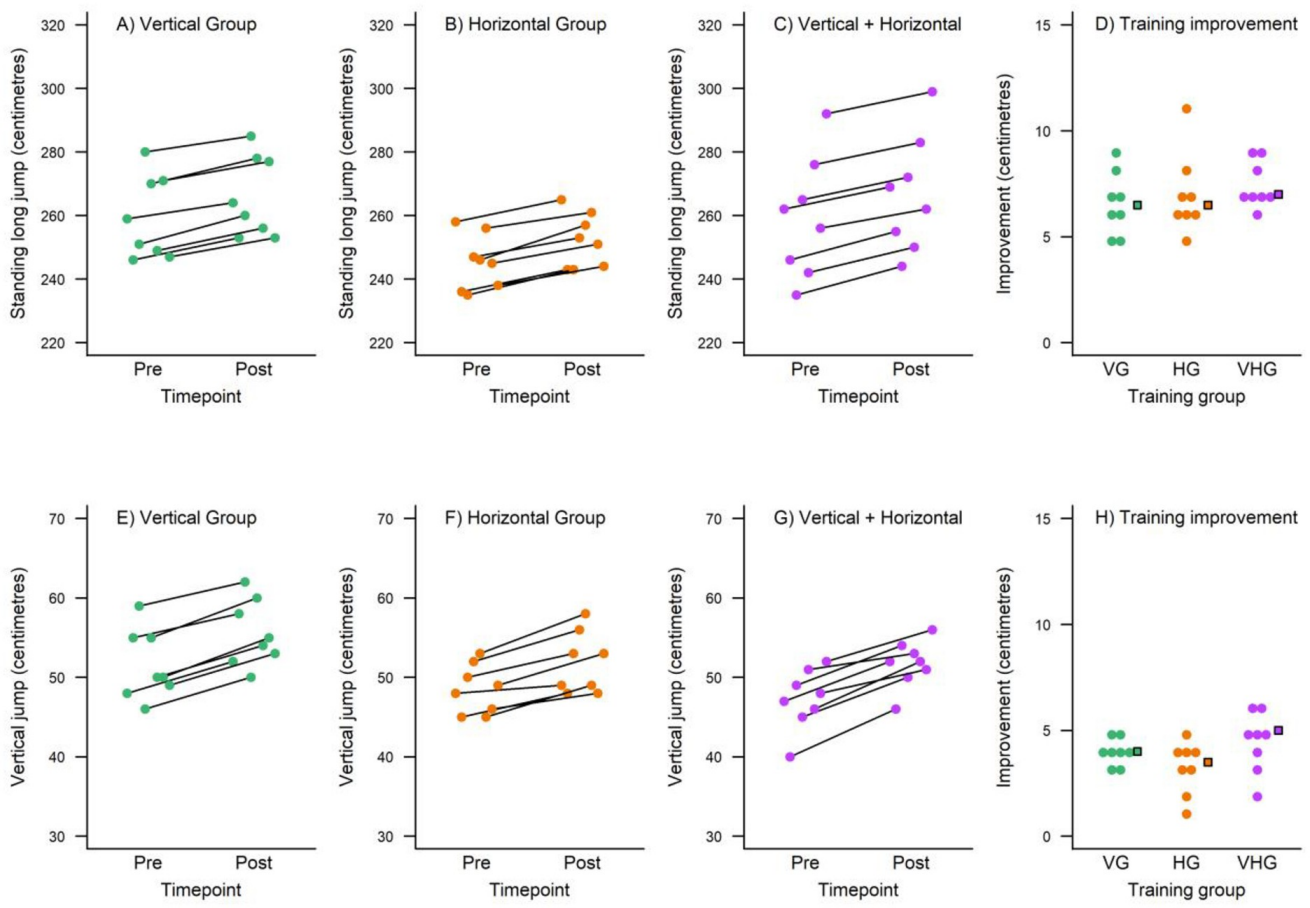
Descriptive plots for the training measures at the Standing long jump (top row) and the Vertical jump test (bottom row) separate for the three training groups (columns). The VG is illustrated in turquoise, HG in orange, and VHG in plum. In the rightmost panels (panel D and H) is the training improvement (*post* minus *pre*) for each individual participant (circles). The median improvement in each training group is also illustrated in the rightmost panels as a square in matching colour.

Fig 1 shows *pre*- and *post*-training measures for each participant for the three sprint tests (separate rows). The rightmost panels in Fig 1 illustrates the improvement, *pre* minus *post*, for each individual participant (circles) and the median improvement in each training group (squares). Fig 2 shows *pre*- and *post*-training measures for the two jumping tests (separate rows) and the rightmost panels illustrate the improvement, *post* minus *pre*. The mean improvements are also listed in Table 2. Effect sizes (Tables 2 and 3) for the 10 m sprint were trivial in every group. A trivial effect size was also observed in the V+HPT group for the 40 m sprint while small effect sizes were observed in the VPT and HPT groups. Moderate effects of varying size were seen for the 505 COD test with the largest in the V+HPT group and the smallest in the VPT group. Largely equivalent small effects were observed for the SLJ test while the V+HPT group achieved the largest effect size for the vertical jump. The V and H groups demonstrated moderate changes for the vertical jump test.

**Table 2:** Group level gains across each of the performance tests.

	Mean improvement (sd)		
Test	Vertical group	Horizontal group	Vertical/horizontal groups
10 m sprint (s)	0.02 (0.02)	0.01 (0.02)	0.02 (0.03)
40 m sprint (s)	0.04 (0.02)	0.07 (0.03)	0.04 (0.01)
505 COD (s)	0.05 (0.04)	0.07 (0.04)	0.08 (0.05)
Standing long jump (cm)	6.63 (1.41)	7.00 (1.85)	7.50 (1.07)
Vertical jump (cm)	4.00 (0.76)	3.25 (1.28)	4.50 (1.41)

**Table 3:** Group level effect sizes across each of the performance tests.

	Effect size (95% confidence interval)		
Test	Vertical group	Horizontal group	Vertical/horizontal groups
10 m sprint	0.10 (-0.06 to 0.26)	0.09 (-0.07 to 0.25)	0.11 (-0.06 to 0.27)
40 m sprint	0.20 (0.05 to 0.36)	0.32 (0.12 to 0.52)	0.19 (0.04 to 0.34)
505 COD	0.77 (-0.04 to 1.58)	1.03 (0.15 to 1.90)	1.14 (0.23 to 2.05)
Standing long jump	0.48 (0.20 to 0.75)	0.51 (0.22 to 0.79)	0.54 (0.24 to 0.84)
Vertical jump	1.07 (0.41 to 1.73)	0.87 (0.30 to 1.45)	1.21 (0.49 to 1.92)

## Discussion

The results of this study suggest that the 8-week PT interventions exerted discernable positive effects on COD and jump performance with few changes seen in linear sprint speed over distances of ten and forty metres. Though demonstrably effective, no specific one of the three applied programmes enhanced performance to a greater extent than another with only the 40 m sprint for the HPT group (mean difference = 0.07 s [HPT] vs. 0.04 s [VPT] and 0.04 s [V+HPT]) and the vertical jump for the V+HPT group (mean difference = 4.5 cm [V+HPT] vs. 4.0 cm [VPT] and 3.25 cm [HPT]) appearing to deviate from a uniform pattern of group level adaptation across the performance tests. There were no apparent directionally-specific adaptive to responses to any of the applied PT programmes.

The results of a recent meta-analysis [5] suggested that whilst both HPT and VPT were both effective for enhancing jump and sprint performance, HPT was just as effective as VPT in enhancing vertical performance (i.e. vertical jump), but was superior at enhancing horizontal performance (i.e. horizontal jump, or sprint). In this way, HPT would appear to be the more effective training activity as it seems to enhance both horizontally- and vertically-orientated performance to a greater extent than VPT. We set out to obtain data that could further clarify these results and examine the potential effects of a third form of PT that combined jumps in both vertical and horizontal direction. Across three volume-equated PT programmes, we were expectant that a combination of both HPT and VPT (V+HPT) would be the most effective form of training and that this would be reflected by increases in performance across the five tests that were chosen. To the best of our knowledge, only one previous study has compared the effects of VPT vs HPT vs V+HPT on physical performance, with that study being carried out in very young male soccer players, aged 10 to 14 years [11]. We sought to repeat a similar investigation to assess the effects of these types of PT in adult players.

The principle of training specificity suggests that adaptations in a given physical capacity will occur if there is a high degree of similarity between an applied training stimulus and the intended outcome of that stimulus [19]. Accordingly, this means that athletes that are involved in sports that require muscular strength for example, should engage in training activities that can cause increases in muscular strength so as to enhance overall performance and success. This has been suggested to apply in relation to PT with the training specificity principle supporting the use of vertical and horizontal jumps to improve performance in vertically- and horizontally-orientated tasks such as jumping and sprinting respectively [2, 3]. However, a recent meta-analysis [5] that analysed all of the available studies in this domain concluded that the transfer of adaptation from vertically-orientated PT to vertically-orientated fitness tests (i.e. countermovement jump) and horizontally-orientated PT to horizontally-orientated fitness tests (i.e. SLJ or 10 m sprint) was not necessarily as clear as the principle of training specificity might suggest and our current results are broadly supportive of this.

Our results also suggest that whilst the PT had little practical effect on both 10 m and 40 m sprint times, there were moderate, bordering on large, effects on COD performance in the 505 test. Why this pattern of results occurred is unclear as Moran et al. [5] had previously reported moderate effects of both HPT and VPT on horizontally-orientated outcomes which included 5 m and 10 m sprint tests. That investigation did not include any tests of COD but a previous meta-analysis by Asadi et al. [20] reported a moderate effect of 0.96 of PT on performance of this type thus corroborating the results of the current study. The researchers’ suggested mechanisms for these responses were changes in the contractile properties of the involved muscle fibres, improved lower-limb muscle activation, greater intermuscular coordination and excitability of the stretch-reflex as well as changes in muscle architecture and stiffness of various elastic components of musculotendinous tissue. Even more curiously, Moran et al. [5] previously reported that HPT was most effective for improving horizontally-orientated performance when training programmes were longer than seven weeks duration and included more than twelve training sessions per programme. The current intervention satisfied these criteria suggesting that the lack of adaptation in the 10 m and 40 m sprint tests could be due to one of the moderator variables that Moran et al. [5] could not account for which was the intensity of the jumps in each programme. Because different varieties of jump do not have uniform kinematic characteristics and elements such as ground reaction forces, they differ in their intensity [21]. In this way, a high intensity PT programme could share an identical volume with a low intensity PT programme yet each would result in a different level of adaptation following execution. This factor is highly difficult to account for in PT meta-analyses and could be a factor in the lack of change in sprint speed observed in the current programme.

A further notable characteristic of this study was the bilateral nature of the jumps that were included in the PT programme and this could have had an impact on whether or not a transfer of effect from training to performance was achieved. It has previously been reported that a combination of bilateral and unilateral HPT is far more effective than bilateral only for enhancing movement speed [5]. Accordingly, PT programmes should include a combination of single- and double-leg jumps to optimise performance. Though a previous meta-analysis [22] has indicated that bilateral and unilateral PT are equally effective for enhancing movement speed, unilateral training might be relatively more beneficial to increasing activity in key muscles such as the vastus medialis and the gastrocnemius [23]. Moreover, due to the presence of the bilateral deficit and longer muscle action times, greater impulses can be achieved in unilateral PT and this could drive additional adaptation [24]. Accordingly, we recommend that future research should incorporate high intensity jumps and jumps of a unilateral nature into the study design. Similarly, coaches should prescribe multidimensional programmes that address the various different demands of soccer incorporating unilateral, bilateral, horizontal, vertical, cyclical and acyclical jumps into the PT protocol [5, 11, 25].

There are some limitations to this study. Though our objective was to compare the effect of PT programme types and not whether PT itself is effective (this is already well-established [26, 27]), the addition of a control group to the study would have offered further basis for comparison. However, it was not possible to require a portion of the involved players to abstain from training for an extended period of time. The addition of unilateral PT may have served as a more comprehensive programme for the players to complete though its deliberate omission allowed us to partially control for its effect on the chosen performance tests and future studies can now expand upon our findings. The sample size in our study was also relatively small with most studies in PT including around ten participants per group [28]. Despite this, the presented data can be very useful for inclusion in future meta-analyses on this particular topic. Lastly, though we included standardised effect sizes (Table 3), we did so for informational rather than analytical purposes. The analysis of such metrics in this type of descriptive analysis with visual representations of the data would not necessarily be appropriate because the absolute gain in performance (Table 2) due to training is more important than the units of between-individual variability. For a summary of the non-standardised mean differences in pre- and post-intervention performance data, the reader is referred to the study dataset in the S1 Data.

## Conclusion

Based on our results, VPT, HPT, and V+H PT had largely similar effects on SLJ, COD, and sprint performance in adult male soccer players. This was a surprising result given the results of previous studies [5, 11] which indicated a more logical task-specific response to the type of training that was applied in the current intervention. Nevertheless, a total volume of 100 foot contacts per session, twice per week for eight weeks was sufficient to achieve the observed changes though adaptations fell somewhat short of addressing the game-specific demands that are imposed upon soccer players during play. Though jump and COD performance were enhanced, linear sprint performance was largely unchanged so a more complete and intense programme may have been warranted. This could have included jumps such as depth or drop jumps from relatively higher heights (i.e. >60 cm) MENDELEY CITATION PLACEHOLDER 38 as well as jumps that are executed unilaterally as well as bilaterally [5]. This could potentially result in adaptations to both jumping and sprinting, rather than just jumping alone. However, coaches must remain cognisant of the training experience of the player, programming more modest volumes of lower intensity jumps before eventual graduation to higher volumes of higher intensity jumps such as those mentioned above. To summarise, based on these results, we recommend [29] that coaches can programme 200 weekly foot contacts of interchangeable combinations of VPT, HPT and V+H PT, erring on the side of caution by prescribing a multidimensional training stimulus that meets the movement demands of soccer. Moreover, a more intense training stimulus or greater volume could induce larger changes in linear sprint performance.

## Supporting information

S1 Data (XLSX)

S1 Checklist *PLOS ONE* clinical studies checklist. (DOCX)

## References

[1] Izquierdo M, Häkkinen K, Gonzalez-Badillo JJ, Ibáñez J, Gorostiaga EM. Effects of long-term training specificity on maximal strength and power of the upper and lower extremities in athletes from different sports. European Journal of Applied Physiology. 2002;87: 264–271. doi: 10.1007/s00421-002-0628-y 12111288

[2] Randell AD, Cronin JB, Keogh JWL, Gill ND. Transference of strength and power adaptation to sports performance-horizontal and vertical force production. Strength and Conditioning Journal. 2010;32: 1524–1602. doi: 10.1519/SSC.0b013e3181e91eec

[3] Goodwin JE, Cleather DJ. The biomechanical principles underpinning strength and conditioning. Strength and conditioning for sports performance. New York: Routledge; 2016.

[4] Fitzpatrick DA, Cimadoro G, Cleather DJ. The magical horizontal force muscle? A preliminary study examining the “force-vector” theory. Sports. 2019;7: 30–38. doi: 10.3390/sports7020030 30678251 PMC6409580

[5] Moran J, Ramirez-Campillo R, Liew B, Chaabene H, Behm D, García-Hermoso A, et al. Effects of vertically- and horizontally-orientated plyometric training on physical performance: a meta-analytical comparison. Sports Medicine. 2021;51: 65–79. doi: 10.1007/s40279-020-01340-6 32897526

[6] Nonnato A, Hulton AT, Brownlee TE, Beato M. The effect of a single session of plyometric training per week on fitness parameters in professional female soccer players: a randomized controlled trial. The Journal of Strength & Conditioning Research. 2022;36: 1046–1052. doi: 10.1519/JSC.0000000000003591 32519832

[7] Bianchi M, Coratella G, Dello Iacono A, Beato M. Comparative effects of single vs. double weekly plyometric training sessions on jump, sprint and change of directions abilities of elite youth football players. J Sports Med Phys Fitness. 2019;59: 910–915. doi: 10.23736/S0022-4707.18.08804-7 30160086

[8] Nagano A, Komura T, Fukashiro S. Optimal coordination of maximal-effort horizontal and vertical jump motions—A computer simulation study. BioMedical Engineering Online. 2007;6: 20–29. doi: 10.1186/1475-925X-6-20 17543118 PMC1896168

[9] Talukdar K, Harrison C, McGuigan M, Borotkanics R. The Effects of Vertical vs. Horizontal Plyometric Training on Sprinting Kinetics in Post Peak Height Female Student Athletes. International Journal of Strength and Conditioning. 2022;2.

[10] Watkins CM, Gill ND, Maunder E, Downes P, Young JD, McGuigan MR, et al. The Effect of Low-Volume Preseason Plyometric Training on Force-Velocity Profiles in Semiprofessional Rugby Union Players. Journal of Strength & Conditioning Research. 2021;35: 604–615. doi: 10.1519/JSC.0000000000003917 33395182

[11] Ramirez-Campillo R, Gallardo F, Henriquez-Olguin C, Meylan CMP, Martinez C, Alvarez C, et al. Effect of vertical, horizontal, and combined plyometric training on explosive, balance and endurance performance of young soccer players. Journal Of Strength And Conditioning Research / National Strength & Conditioning Association. 2015;29: 1784–1795. doi: 10.1519/JSC.0000000000000827 25559903

[12] Bizzini M, Dvorak J. FIFA 11+: an effective programme to prevent football injuries in various player groups worldwide—a narrative review. Br J Sports Med. 2015;49: 577–579. doi: 10.1136/bjsports-2015-094765 25878073 PMC4413741

[13] Fernandez-Santos JR, Ruiz JR, Cohen DD, Gonzalez-Montesinos JL, Castro-Piñero J. Reliability and Validity of Tests to Assess Lower-Body Muscular Power in Children. The Journal of Strength & Conditioning Research. 2015;29: 2277–2285. doi: 10.1519/JSC.0000000000000864 25647647

[14] Nobari H, Silva AF, Vali N, Clemente FMM. Comparing the physical effects of combining small-sided games with short high-intensity interval training or repeated sprint training in youth soccer players: A parallel-study design. International Journal of Sports Science & Coaching. 2022;In press.

[15] Enoksen E, Tønnessen E, Shalfawi S. Validity and reliability of the Newtest Powertimer 300-series® testing system. Journal of Sports Sciences. 2009;27: 77–84. doi: 10.1080/02640410802448723 19031330

[16] Nobari H, Cholewa JM, Castillo-Rodríguez A, Kargarfard M, Pérez-Gómez J. Effects of chronic betaine supplementation on performance in professional young soccer players during a competitive season: a double blind, randomized, placebo-controlled trial. Journal of the International Society of Sports Nutrition. 2021;18: 1–12. 34663363 10.1186/s12970-021-00464-y PMC8525016

[17] Sand A. Inferential Statistics Is an Unfit Tool for Interpreting Data. Applied Sciences. 2022;12.

[18] Wasserstein RL, Schirm AL, Lazar NA. Moving to a World Beyond “p < 0.05”. The American Statistician. 2019;73: 1–19.

[19] Hawley JA. Specificity of training adaptation: time for a rethink? Journal of Physiology. 2008;586: 1–2. doi: 10.1113/jphysiol.2007.147397 18167367 PMC2375570

[20] Asadi A, Arazi H, Young WB, de Villarreal ES. The effects of plyometric training on change-of-direction ability: A meta-analysis. International Journal of Sports Physiology and Performance. 2016;11: 563–573. doi: 10.1123/ijspp.2015-0694 27139591

[21] Jarvis MM, Graham-Smith P, Comfort P. A Methodological Approach to Quantifying Plyometric Intensity. Journal of Strength & Conditioning Research. 2016;30: 2522–2532. doi: 10.1519/JSC.0000000000000518 24787677

[22] Moran J, Ramirez-Campillo R, Liew B, Chaabene H, Behm D, García-Hermoso A, et al. Effects of Bilateral and Unilateral Resistance Training on Horizontally-Orientated Movement Performance: A Systematic Review and Meta-Analysis. Sports Medicine. 2021;51: 225–242. doi: 10.1007/s40279-020-01367-9 33104995

[23] Bogdanis GC, Tsoukos A, Kaloheri O, Terzis G, Veligekas P, Brown LE. Comparison between unilateral and bilateral plyometric training on single- and double-leg jumping performance and strength. Journal of Strength and Conditioning Research. 2019;33: 633–640. doi: 10.1519/JSC.0000000000001962 28445230

[24] Bobbert MF, De Graaf WW, Jonk JN, Casius LJR. Explanation of the bilateral deficit in human vertical squat jumping. Journal of Applied Physiology. 2006;100: 493–499. doi: 10.1152/japplphysiol.00637.2005 16239616

[25] Ramírez-Campillo R, Meylan CM, Álvarez-Lepín C, Henriquez-Olguín C, Martinez C, Andrade DC, et al. The effects of interday rest on adaptation to 6 weeks of plyometric training in young soccer players. The Journal of Strength & Conditioning Research. 2015;29: 972–979. 10.1519/JSC.0000000000000283 24149761

[26] de Villarreal ES, Requena B, Cronin JB. The effects of plyometric training on sprint performance: A meta-analysis. Journal of Strength and Conditioning Research. 2012;26: 575–584. doi: 10.1519/JSC.0b013e318220fd03 22240550

[27] de Villarreal ES-S, Kellis E, Kraemer WJ, Izquierdo M. Determining variables of plyometric training for improving vertical jump height performance: a meta-analysis. Journal of strength and conditioning research / National Strength & Conditioning Association. 2009;23: 495–506. doi: 10.1519/JSC.0b013e318196b7c6 19197203

[28] Ramirez-Campillo R, Moran J, Chaabene H, Granacher U, Behm DG, García-Hermoso A, et al. Methodological characteristics and future directions for plyometric jump training research: A scoping review update. Scandinavian Journal of Medicine and Science in Sports. 2020;30: 983–997. doi: 10.1111/sms.13633 32034819

[29] Wallace BJ, Kernozek TW, White JM, Kline DE, Wright GA, Peng HT, et al. Quantification of Vertical Ground Reaction Forces of Popular Bilateral Plyometric Exercises. Journal of Strength & Conditioning Research. 2010;24: 207–212. doi: 10.1519/JSC.0b013e3181c3b841 19924006

