# Supplementary material for: Effect of vertical, horizontal, and combined plyometric training on jump, sprint and change of direction performance in male soccer players
Source: PLoS One. 2024 May 23;19(5):e0295786. doi: 10.1371/journal.pone.0295786 (PMC11115329; doi:10.1371/journal.pone.0295786)
Supplement: S1 Checklist — (DOCX) [file pone.0295786.s002.docx]

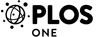


***PLOS ONE* Clinical Studies Checklist**

*PLOS ONE* manuscript number: PONE-D-22-27284

| ***Complete the following if your study involved human participants or human subjects’ data.***  ***These questions should be addressed for prospective and retrospective studies.*** | |
| --- | --- |
| 1. | Did you obtain ethics approval for this study?   - If yes, please upload (file type “Other”) the original approval document you received from your ethics committee. If the original document is in another language, please also provide an English translation.   _√_ Uploaded N/A   - If you did not obtain ethical approval, please explain why this was not required. |
|  |  |
| 2. | If your study involved human participants, please report in the Methods section when participants were recruited to the study.  _*√_* Completed N/A |
| 3. | If you are reporting a study of medical records or archived samples, please report in the Methods section the date range in which human subjects’ data/samples were collected and the date(s) when you conducted this study.  __ Completed √N/A |
| 4. | Please specify in the Methods section whether authors had access to information that could identify individual participants during or after data collection.  __ Completed √N/A |
| 5. | If you are reporting an observational study – i.e. cohort, case-control, and cross-sectional studies  – we recommend that the work is reported as per the requirements of the STROBE guidelines, and that you provide a completed STROBE checklist as a Supporting Information file with your submission.  The STROBE checklist was developed to improve the reporting of observational human subjects research, and is available here: [http://strobe-](http://strobe-statement.org/fileadmin/Strobe/uploads/checklists/STROBE_checklist_v4_combined_PlosMedicine.docx) [statement.org/fileadmin/Strobe/uploads/checklists/STROBE_checklist_v4_combined_PlosMedici](http://strobe-statement.org/fileadmin/Strobe/uploads/checklists/STROBE_checklist_v4_combined_PlosMedicine.docx) [ne.docx.](http://strobe-statement.org/fileadmin/Strobe/uploads/checklists/STROBE_checklist_v4_combined_PlosMedicine.docx)  Completed _ √_ N/A |
| 6. | Please ensure that the author list and Corresponding Author entered in Editorial Manager match the author list and Corresponding Author in your manuscript file.  _√_ Completed N/A |

Last Modified March 2016
